# P04.74. Use of mind-body therapies among adults with neuropsychiatric symptoms common to mild traumatic brain injury

**DOI:** 10.1186/1472-6882-12-S1-P344

**Published:** 2012-06-12

**Authors:** M Purohit, S Bertisch, R Wells, R Zafonte, R Phillips

**Affiliations:** 1 Beth Israel Deaconess Medical Center/Harvard Medical School, Boston, USA; 2 Brigham and Women's Hospital/Faulkner Hospital/Harvard Medical School, Boston, USA; 3 Spaulding Rehabilitation Hospital/Harvard Medical School, Boston, USA

## Purpose

Neuropsychiatric symptoms caused by mild Traumatic Brain Injury (mTBI) are difficult to treat with standard interventions. Given such limitations, patients may choose to treat themselves with mind-body therapies. However, little is known about the use of mind-body therapies by adults with neuropsychiatric symptoms associated with mTBI.

## Methods

We compared mind-body therapy use between adults with and without neuropsychiatric symptoms associated with mTBI (self reported anxiety, depression, insomnia, headaches, memory deficits, attention deficits, and excessive daytime sleepiness) using the 2007 National Health Interview Survey (n=23,393). Mind-body therapy use was defined as use of ≥1 therapy of meditation, yoga, acupuncture, deep-breathing exercises, hypnosis, progressive relaxation therapy, qi gong, and tai chi within the past year. We examined prevalence and reasons for mind-body therapy use in adults with neuropsychiatric symptoms and explored variations in use by number of symptoms. We performed logistic regression to examine the association between neuropsychiatric symptoms and mind-body therapy use, after adjusting for sociodemographic characteristics, illness burden, access to care, and health habits.

## Results

Adults with ≥1 neuropsychiatric symptoms used mind-body therapy more than adults without symptoms (25.8% vs. 15.3%, p<0.001). Prevalence increased with increasing number of symptoms (22.1% for 1 symptom, 31.8% for ≥3 symptoms, p<0.001); differences persisted after adjustment (aOR 1.38 [1.25, 1.52] and 2.10 [1.83, 2.41], respectively, compared to adults without symptoms). Reasons for mind-body therapy use among adults with ≥1 symptom include general wellness (64.4%), conventional medicine was ineffective or too expensive (30.2%), and conventional provider recommendation (27.8%). Seventy percent of adults with ≥1 symptom did not discuss their mind-body therapy use with a conventional provider.

## Conclusion

More than one in four adults with ≥1 neuropsychiatric symptom used mind-body therapies, with more symptoms associated with increased use. Future research is needed to understand the efficacy and cost of mind-body therapies for patients with neuropsychiatric symptoms common to mTBI.

